# Three novel variants identified within ECM-related genes in Chinese Han keratoconus patients

**DOI:** 10.1038/s41598-020-62572-0

**Published:** 2020-04-03

**Authors:** Xiayan Xu, Xin Zhang, Yilei Cui, Hao Yang, Xiyuan Ping, Jing Wu, Xiaoning Yu, Xiuming Jin, Xiaodan Huang, Xingchao Shentu

**Affiliations:** Department of Ophthalmology, The Second Affiliated Hospital of Zhejiang University, College of Medicine, Hangzhou, Zhejiang China

**Keywords:** Gene expression, Next-generation sequencing

## Abstract

As the primary indication for corneal transplantation, the pathogenesis of keratoconus remains elusive. Aiming to identify whether any mutation from extracellular-matrix (ECM)-related genes contributes to the patients with sporadic cases of keratoconus (KC) from Chinese Han population, one hundred and fifty-three participants in total were enrolled in our study, including fifty-three KC patients and one hundred healthy controls. Mutational analysis of three ECM-related genes (*LOX*, *COL5A1* and *TIMP3*) with next-generation sequencing and Sanger sequencing was performed. To further confirm the function of three ECM-related genes in the pathogenesis of keratoconus, we performed Real-time Quantitative PCR *in vitro*. Results showed that three new sequence variants (c.95 G > A in *LOX*, c.1372 C > T in *COL5A1* and c.476 C > T in *TIMP3*) were identified in aforementioned ECM-related genes in KC patients without being detected among the healthy controls. According to the results of QPCR, we found that the expression levels of *LOX* and *TIMP3* were decreased in the KC patients, while *COL5A1* showed no significant difference of expression. This is the first time to screen so many ECM-related genes in Chinese keratoconus patients using next-generation sequencing. We find numerous underlying causal variants, enlarging lots of mutation spectrums and thus providing new sites for other investigators to replicate and for further research.

## Introduction

Keratoconus (KC) is a progressive disorder characterized by central cornea thinning and ectasia in a cone-shape fashion, leading to myopia, irregular astigmatism and even vision loss^1,2^. KC usually occurs from the second decade to the fourth decade of life, and affects both genders. The prevalence of KC ranges from 900 to 3300 per 100,000 in recent population-based studies^2^. Clinically, corneal tomography is most frequently used for diagnosis of KC, while mild or subclinical KC can be diagnosed by posterior corneal elevation abnormalities^3^. The treatments of KC often begin with verbal guidance such as not rubbing eyes and wearing contact lenses, with 10–20% of patients finally turning to corneal transplantation^3,4^. Collagen cross-linking (CXL) is a novel intervention effective in the therapy of KC.

However, the exact etiology and pathogenesis of KC remains unclear, in which genetic, environmental, biomechanical and biochemical factors may involve^3^. Eye rubbing^5^, atopy^6,7^ and sun exposure^2^ are important environmental factors indicating a high risk of KC. In addition, there are increasing evidences suggesting a genetic predisposition in the pathogenesis of keratoconus, with lots of genomic loci and genes identified, including visual system homeobox 1 (VSX1)^8–10^, superoxide dismutase 1 (SOD1)^10,11^, transforming growth factor beta-induced (TGFβI)^12^ and microRNA 184 (MIR184)^13,14^. Nevertheless, it is to be further explored whether and how these genomic loci and genes participate in the progression of KC.

As a major component of the cornea, the corneal stroma rich in extracellular matrix (ECM) plays an important role in cornea diseases, thinning of which cannot resist normal intraocular pressure, causing cornea protruding and finally developing KC. Over years, studies on the relationship between ECM and KC have been more and more conducted, and many ECM-related genes and corresponding proteins have been found to be potentially involved in the pathogenesis of KC, such as glycoprotein fibronectin (FN1)^15^, integrin^15^, metalloproteinase (MMP9)^15,16^, tissue inhibitor of metalloproteinase (TIMP1, TIMP2)^15,16^, thrombospondin1 (THBS1)^15,17^, transforming growth factor beta-induced gene (TGFBI)^15,18,19^, *et al. LOX*, *COL5A1* and *TIMP3* were three ECM-related genes identified in this study. The *LOX* gene is located on the 5q23.2 chromosomal region, including seven exons and six introns^20^. The inactive 50 kDa pro-enzyme is first produced until processed by pro-collagen C-proteinases—mammalian Tolloids and bone morphogenetic protein-1 (BMP-1) to become active enough to cross link collagens and elastin by catalyzing oxidative deamination of peptidyl lysines^20–23^. The *COL5A1* gene, located on 9q34.2, encodes the α1 chain of type V collagen, which regulates collagen fibrillogenesis^24,25^. Collagen V is a quantitatively minor component in most tissues, and often functions in a heterotypic form with collagen I^26,27^. Collagen V has different isoforms, of which the most abundant and ubiquitous form is the heterotrimer [α1(V)]_2_α2(V), then α1(V)α2(V)α3(V) and [α1(V)]_2_α4(V), with [α1(V)]_3_ homotrimer the least common form showing a more restricted expression pattern^28^. The high proportion of type V collagen in the cornea leads to the large number of nucleation sites, which may account for the great number and small diameter of fibril necessary for transparency^29,30^. The *TIMP3* gene is on 22q12.3, containing 5 exons. The gene products TIMP3 is a tissue specific, endogenous inhibitor of metalloproteinase (MMP), thus playing an important role in extracellular matrix remodeling and potentially KC progression^31,32^.

In this study, we aimed to make further explorations on the biomechanical nature of KC, and sequenced several ECM-related genes in a Chinese Han population by next-generation sequencing. According to the results of sequencing, we found that three variants in three genes respectively (c.95 G > A in *LOX*, c.1372 C > T in *COL5A1* and c.476 C > T in *TIMP3*) might play a role in the pathogenesis of keratoconus. Further QPCR conduction showed that the expression levels of *LOX* and *TIMP3* were decreased in the KC patients, while *COL5A1* showed no significant difference of expression between KC and healthy controls.

## Results

**Figure 1:**
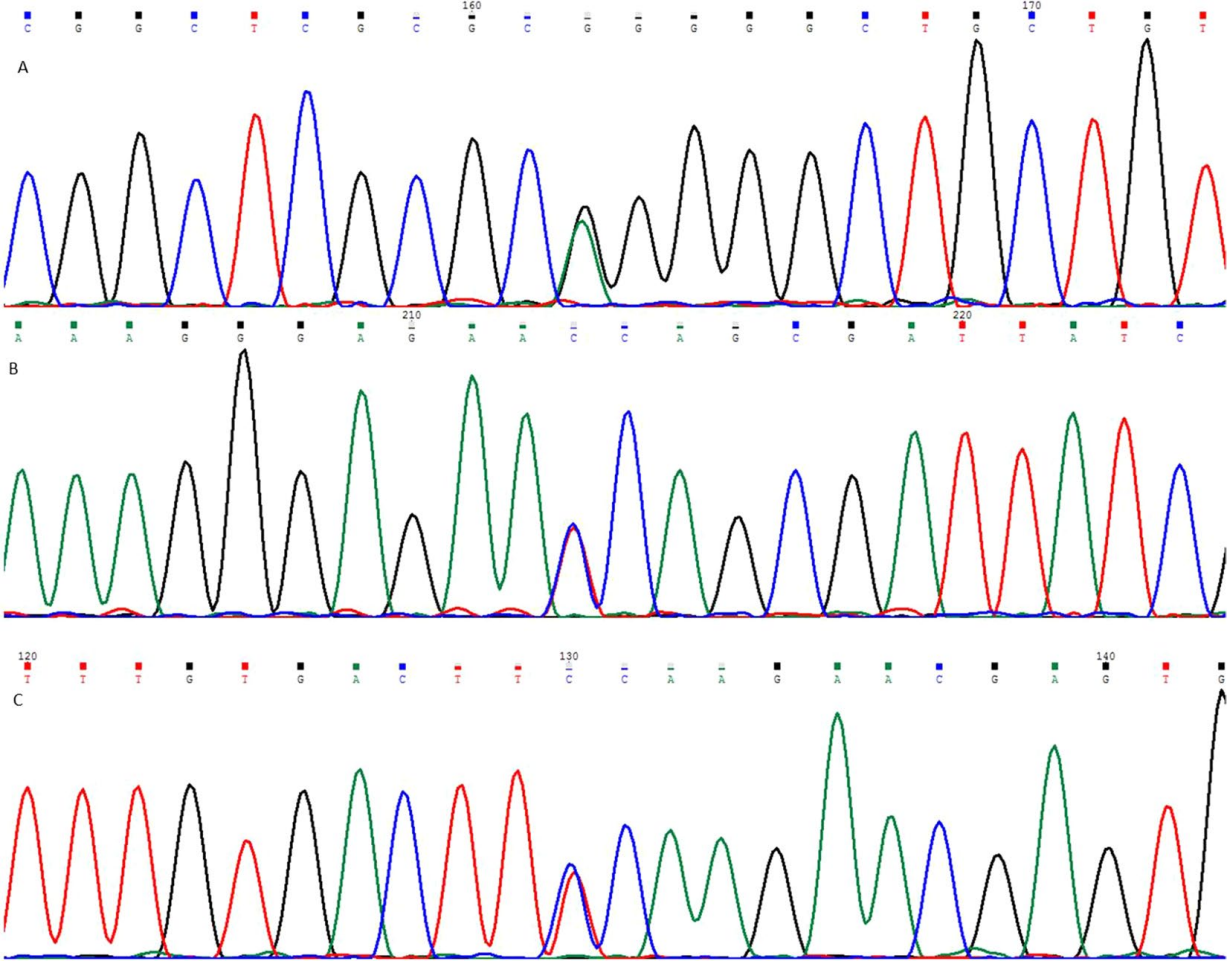
Sequence chromatograms of LOX (**A**), COL5A1 (**B**) and TIMP3 (**C**).

A total of 53 keratoconus (KC) patients and 100 healthy controls were included in this study. Demographic characteristics of all KC patients and three patients carrying target variants were shown in Table 1 and Table 2 respectively. According to the results of next-generation sequencing in KC patients, three single nucleotide variants were separately identified in three extracellular-matrix related genes (c.95 G > A in *LOX*, c.1372 C > T in *COL5A1* and c.476 C > T in *TIMP3*). Sanger sequencing were then conducted in 100 healthy controls to rule out the possibility of false positives. Sequencing chromatograms of the three mutations were shown in Fig. 1, and all were located in the exon regions of the corresponding genes. None of the three mutations were classified as tolerated according to SIFT (Table 3).

**Table 1:** The general demographic characteristics of the 53 keratoconus patients in this study.

Demographic characteristics		
Sex	Males	41 (77.36%)
	Females	12 (22.64%)
Age		27.04 ± 6.35
Age at diagnosis (year)		20.06 ± 4.18
Disease laterality	OD	4 (7.55%)
	OS	6 (11.32%)
	OU	43 (81.13%)
Diopter (OD)		−6.78 ± 4.14
Diopter (OS)		−6.14 ± 3.4
Corneal transplantation history	Positive	1 (1.89%)
	Negative	52 (98.11%)
Corneal thickness (OD, at the thinnest point)		461.45 ± 56.64
Corneal thickness (OS, at the thinnest point)		471.47 ± 50.7

**Table 2:** The demographic characteristics of the 3 keratoconus patients carrying target mutations in this study.

Mutation	Sex	Age	Age at diagnosis	Disease laterality	Diopter (OD)	Diopter (OS)	Corneal transplantation history	Corneal thickness (OD)	Corneal thickness (OS)
c.95 G > A in *LOX*	Male	28	21	OU	−6.5	−5.25	negative	467	489
c.1372 C > T in *COL5A1*	Male	32	25	OU	−5.75	−11.5	negative	501	468
c.476 C > T in *TIMP3*	Male	20	15	OU	−4.75	−9.5	negative	472	444

**Table 3:** Three novel mutations respectively of *LOX*, *COL5A1* and *TIMP3* identified in KC patients.

Gene	Nucleotide change	Amino acid change	Position	Gene region	Mutation effect	SIFT score
*LOX*	c.95 G > A	P32L	121413586	exonic	nonsynonymous SNV	0.01
*COL5A1*	c.1372 C > T	S159F	33255204	exonic	nonsynonymous SNV	0
*TIMP3*	c.476 C > T	P458S	137623956	exonic	nonsynonymous SNV	0.01

**Figure 2:**
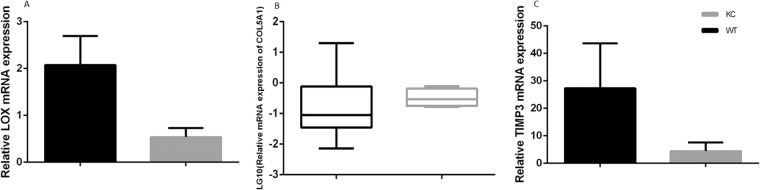
Relative mRNA expressions of LOX (left, **A**), COL5A1 (middle, **B**) and TIMP3 (right, **C**) in the KC patients (grey panels) and healthy controls (black panel).

Based on the results of sequencing, QPCR was further conducted to explore the molecular manifestations of aforementioned three genes in 6 KC patients and 4 healthy controls. There were of no significant difference on demographic characteristics between KC and control groups (Table 4). Results of QPCR (Fig. 2) showed that mRNA expression of *LOX* and *TIMP3* were significantly higher in KC corneas compared to controls (P < 0.01 and P = 0.0297 respectively), while no significant differences were observed on *COL5A1* expression (P = 0.6252).

**Table 4:** Demographic characteristics of 6 keratoconus patients and 4 healthy controls for QPCR experiment.

Group	Sex (%)		Age (year)
Keratoconus patients	Males	66.7%	44.67 ± 18.02
	Females	33.3%	
Healthy controls	Males	75%	53.75 ± 13.7
	Females	25%	
P value	0.807		0.419

## Discussion

In this study, three novel mutations (c.95 G > A in *LOX*, c.1372 C > T in *COL5A1* and c.476 C > T in *TIMP3*) leading to the following amino acid substitutions P32L, P458S and S159F, were recognized in a Chinese Han population of 53 keratoconus (KC) patients. All mutations were discovered in sporadic cases by next generation sequencing and validated in 100 healthy controls by Sanger sequencing and identified as damaging according to the results of SIFT. SIFT is an online tool distinguishing damaging amino acid substitutions from tolerant ones, which is based on sequence homology and the severity of the corresponding amino acid change^18,19^. The outstanding advantage of SIFT is not requiring structure but having similar power to those that use structure^20,21^. Further QPCR performance showed a decreased mRNA expression of *LOX* and *TIMP3* in KC corneas, with no significant discrepancy found in *COL5A1* expression.

KC is a multifactorial disease with the exact etiology remaining unclear to date. Stromal thinning is an important hallmark of KC, with more studies focusing on the mechanisms of biomechanical factors in the pathogenesis of KC^33–35^. In our study, *LOX*, *COL5A1* and *TIMP3* were three ECM-related genes playing different roles in the pathogenesis of KC. First, COL5A1 is a component of ECM regulating collagen fibrillogenesis^24,25^. Then, LOX catalyzes the formation of covalent bonds between elastin and collagens which promotes the maturation of ECM^36–38^. Finally, TIMP3 works to resist the function of MMP or facilitate the apoptosis of stromal cells, thus playing a unique role in ECM remodeling^38^. These all implicated that instability of ECM may be a crucial mechanism underlying the pathogenesis of KC.

The *LOX* gene encodes a copper-dependent amine oxidase which is crucial for cross-linking of ECM, the stability of which is thus guaranteed^39^. Recently, more and more studies have shown LOX as a candidate gene for KC^38–46^. Multiple *LOX* mutations have been detected in sporadic (rs1800449 in the Iranian population^40^, rs2956540 in the Chinese^44^ and European^45^ population) and familial (rs2956540, rs10519694 rs1800449 and rs2288393 in the American population^46^) cases. Molecular evidences further showed a positive correlation between decreased expression and activity of *LOX* and the severity of KC^41,42^. In our study, we found a new mutation (c.95 G > A) in *LOX*, accompanied by a reduction in *LOX* mRNA expression in KC patients. It can be well explained that destruction of normal LOX expression destroys ECM maturation by reduced cross-linking of collagen fibers in the cornea stroma, leading to cornea biomechanical instability and thinning^31,47^, which is a remarkable characteristic of KC. It can be further proved by observing reduced crosslinks between collagen and elastic fibers in *LOX*-null mice^48,49^. So it is a reasonable guess that normal *LOX* expression in the cornea is important in stabilizing the structure and function of the cornea; mutations in *LOX* may affect expression of corresponding proteins by alternative splicing^42^. Collagen cross linking (CXL) is a relatively safe and well-tolerated treatment to KC^50^, the principle of which is similar to the function mechanism of LOX. The finding that *LOX* was higher expressed in the high-response-to-CXL group compared to the low response group^51^ further confirmed the function of LOX and its potential role in KC. However, some SNPs (rs2956540^44–46^, rs10519694^46^, rs1800449^40,46^ and rs2288393^46^) confirmed in previous studies was undetected in this study, which may be due to the limited number of patients and different populations. Otherwise, a study in 2012 even did not find any pathogenic variant in KC^38^, and another gene expression microarray study showed a oppositely increasing trend in the KC patients compared to the normal controls^52^. These all indicated the elusive pathogenic mechanisms of KC in which LOX may include various mutants and play multiple roles by different signal pathways.

*COL5A1* is a kind of central-cornea-thickness (CCT)-related gene that codes for an alpha chain of Collagen V^53^. Many population studies have found that *COL5A1* was associated with corneal thinning^54–56^, which is characteristic of KC. SNPs rs7044529 and rs1536482 of *COL5A1* were also indicated to be related with KC in several population studies^53,54,57^. However, some subsequent analyses of KC population showed no significant difference of *COL5A1* minor allele frequency (MAF) between KC patients and controls^40,44^, making the function of *COL5A1* in KC confusing. In our study, a new mutation in *COL5A1* (c.1372 C > T) was detected. However, the result of QPCR that the mRNA expression was of no significant difference between KC patients and healthy controls (P = 0.6264) was beyond expectation. We speculate that *COL5A1* may be a potentially pathogenic locus for KC as verified in many previous studies^53,58^, but not all KC patients carry the mutation of this gene, perhaps related with race, region and so on. In addition, although the expression level of *COL5A1* in KC was comparable with that in the control, the structure or function of that protein might have been damaged in the KC group, thus also contributing the development of KC. As for the discrete mechanisms, it is well expected that the different compositions of Collagen V may play a role in the pathogenesis of KC as α1 homotrimer being the predominant form is not able to be well incorporated into ECM, finally destroying the integrity and stability of ECM^59^. The construction of conditional-col5α2-knock-out mice model verified this opinion from the other perspective^29^. The targeted deletion of col5α2 caused the homotrimer [α1(V)]_3_ the major form which is unable to be absorbed into the heterotypic collagen fibrils, thus impairing skin matrix organization^59^. Another murine model also showed that the cornea was thinner and had fewer collagen fibrils in heterozygous col5α1 null mice than in wild type mice^30,60^.

The protein product of gene *TIMP3* is a type of tissue inhibitor of metalloproteinase (TIMP), which functions against matrix metalloproteinase (MMP) to protect tissues from irreversible destruction^32,61^. The capacity of TIMP3 on ECM remodeling makes it a candidate for KC progression. However, few studies focused on the relationship between TIMP3 and KC, and no pathogenic variants have been found so far^32^. As for molecular findings, different studies showed contradictory results^38,62^. Ji-Eun Lee *et al*.^62^ found that TIMP3 was underexpressed in KC patients compared to controls, while Matthews *et al*. represented a high expression of TIMP3 and active apoptosis in KC corneas^38^. In our study, we discovered a new mutation (c.476 C > T) in *TIMP3*, and found a decreased expression of *TIMP3* in KC patients. It was expected that TIMP3 functioned as an inhibitor of MMP in the healthy cornea, and disruption of its normal structure or function caused itself unable to protect tissues from irreversible destruction of extracellular matrix^62^, finally turning to KC. However, previous studies have shown that TIMP3 could easily trigger apoptosis of neighboring cells when in a matrix-bound and high-concentration form. Therefore, concurrent detections of apoptosis markers and *TIMP3* expression might further distinguish the comprehensive functions of TIMP3 in KC.

In conclusion, this study discovered three novel variants in three ECM-related genes respectively in the Chinese Han population (c.95 G > A in *LOX*, c.1372 C > T in *COL5A1*, and c.476 C > T in *TIMP3*), and the results of QPCR indicated that the abnormally low expression of *LOX* and *TIMP3* might contribute to the development of KC, all these highlighting the importance of ECM in the pathogenesis of KC. The result that the expression of *COL5A1* was of no significant difference between the control and KC group, did not negate the potential role of COL5A1 in the pathogenesis of KC, but indicated the complex mechanisms underlying KC among different races, regions, and so on. Besides, change of function is as important as change of the expression level in the pathogenesis of diseases, so the additional detection of COL5A1 function by mutation screening and the SIFT score or other methods may better translate the result of QPCR. This study enlarges KC-related mutation spectrums and the novel mutations found here can be used for further validation and research, making a deep understanding of KC and thus contributing to the development of KC therapy. However, Because of the limited samples obtained in this study, further larger and multi-center population studies need to be taken to confirm the danger of these variants as well as functional experiments to deep dig into the nature of KC.

## Methods

The study was conducted in accordance with the tenets of the Declaration of Helsinki and approved by the ethics committee of Second Affiliated Hospital, Medical College of Zhejiang University, Hangzhou, China. Written informed consent was obtained from all participating individuals or their guardians after explanation of possible consequences of the study.

### Study participants

Totally, fifty-three clinically affected isolated keratoconus patients of Chinese Han ethnicity and one hundred unrelated population-matched healthy controls without any ocular or systemic disorders were recruited from Eye Center of Second Affiliated Hospital, Medical College of Zhejiang University, during the period of 2013 to 2015. Following thorough inquiry, negative family histories taken, each participate underwent a comprehensive ocular and systemic evaluation. Any keratoconus cases with co-existing allergy/atopy or secondary to causes such as trauma, Laser-Assisted *in situ* Keratomileusis (LASIK) or other refractive surgeries, Ehlers Danlos syndrome, Down syndrome, Osteogenesis Imperfecta and pellucid marginal degeneration were excluded from the study.

The diagnosis of keratoconus was carried out by an experienced ophthalmologist based on key features exhibited through slit-lamp biomicroscopy, cycloplegic retinoscopy, and corneal topography^63^. Slit-lamp biomicroscopy was used to identify well-established clinical signs of keratoconus including stromal corneal thinning, Vogt’s striae and Fleischer rings in participants. The oil droplet sign and scissoring of the red reflex were assessed by retinoscopy performed with a fully dilated pupil. Patients were considered keratoconus if they had at least one clinical sign accompanied with a confirmatory videokeratography map^63^. The detailed criterion selected in this article was posterior corneal elevation ≥ +20 um within the central 5 mm and inferior-superior dioptric asymmetry (*I-S* value)> 1.2 diopters (D), with the steepest keratometry > 47D^13^.

### Mutation screening

Peripheral blood samples of all above-mentioned participants were collected in Vacutainer tubes (Becton-Dickinson, Franklin Lakes, NJ, USA) containing ethylene diamine tetraacetic acid (EDTA) and genomic DNA was isolated using the Simgen Blood DNA mini kit (Simgen, Hangzhou, China)^64^. Mutation screening was performed using genomic DNA samples from affected participants as well as healthy controls. For patients, several extracellular-matrix (ECM)-related genes suggested involved in keratoconus were screened by next-generation sequencing and confirmed by directly sequencing. Subsequently, probable pathogenic variants were analyzed in a healthy control population using Sanger sequencing analysis. All coding regions comprised of all exons, intron-exon junctions and promoter regions of the candidate genes were amplified by polymerase chain reaction (PCR) using specific primer sequences. The PCR products were isolated by electrophoresis on 1.0% agarose gels and sequenced with the BigDye Terminator Cycle sequencing kit V3.1 (Applied Biosystems, Foster City, CA) on an Applied Biosystems ABI3730 Sequence Analyzer. The sequencing results were analyzed using Polyphred and compared with the sequences in the NCBI GenBank database^1^.

### Bioinformatics analysis

To predict the effect of this amino acid substitution on the protein, we used the online tools SIFT (Sorting Intolerant Form Tolerant, http://sift.jcvi.org/) programs. Using structural and comparative evolutionary considerations, the prediction result of SIFT ranges from 0 to 1 based on evolutionary conservation. The amino acid substitution is predicted damaging if the score is ≤0.05, and tolerated if the score is >0.05.

### Isolation of RNA, cDNA synthesis, and real-time QPCR

Total corneas were collected from 6 keratoconus patients and 4 healthy controls who were met with the aforementioned criteria listed in the Study Participant part. Debrided cells were immediately transferred to −80 °C for storage until processing for RNA extraction.

Total RNA was extracted using TRIzol reagent (Invitrogen), and reverse transcription was performed with ReverTra Ace (Toyobo, Osaka, Japan) according to the manufacturer’s instructions. Real-time QPCR was performed on a Light-Cycler Roche480 (Roche Molecular Systems) using the SYBR Green Master Kit (Bimake). The mRNA levels were calculated using the ΔΔCt method. All qPCR primers and their sequences were as follows: *LOX* (5-CTTGCACGTTTCCAATCGCA-3, 5-ATGCCAAGGGTGGGATTCAG-3), *COL5A1* (5-ACGGGAATGGCGAGAACTAC-3, 5-GAGCAGTTTCCCACGCTTGA-3), *TIMP3* (5-ACCGAGGCTTCACCAAGATG-3, 5-CAGGGGTCTGTGGCATTGAT-3), and *GADPH* (5-GAATGGGCAGCCGTTAGGAA-3, 5-AAAAGCATCACCCGGAGGAG-3).

### Statistical analysis

All results were expressed as the mean ± S.D. The p value was calculated using the GraphPad Prism version 5 statistical program and determined by two-tailed Student’s t test (*LOX*, lg10 (*COL5A1*) and *TIMP3*). A value of p < 0.05 was considered statistically significant.

## Data Availability

Readers are welcome to comment on the online version of the paper. All data included in this study are available upon request by contact with the corresponding author Xingchao Shentu (stxc@zju.edu.cn).
